# What about Dr. Koch, and What about Intensely Speculative Medicine?

**Published:** 1891-05

**Authors:** E. F. Starr

**Affiliations:** Nacoochee, Ga.


### WHAT ABOUT DR. KOCH, AND WHAT ABOUT INTENSELY SPECULATIVE MEDICINE?

By E. F. STARR, M. D., Nacoochee, Ga.

Professor Virchow, of Berlin, it seems has put his foot down rather emphatically on Koch's lymph—this world-renowned new discovery. He (Virchow) has said, in substance, it does not cure consumption, but produces inflammations in other parts.

I am not at all amazed at this announcement. It is about as much as I had expected. I had not been enthusiastic on this little matter that has kindled so great a fire in the world, medical and lay, or professional and lay, as I had but little faith. So we will have to try again. The siege shall still go on, but the approaches will have to be made from some other direction or constructed in a different way. The lymph, from what I can learn, appears to be a ptomaine composed of different bacilli and glycerine, and is about as apt to be destructive to the *genus homo* as to the famed bacillus; and even if it were to destroy the microbe and leave the man intact, consumption would still exist and still go on and kill the patient all the same, unless something more could be done—some change were made, some revolution wrought, in the intrinsic status of the organism. The enemy is strongly ensconced, and the capture of a few stragglers will not dislodge him nor put an end to the struggle.

Except for the fact that it has been well conceded that enough germicides could not be introduced into the human system to destroy the microbes without jeopardizing the patient, Koch's device might be considered an ingenious one, but as it is, it was hardly so. He found, it seems, that the ptomaine of dead bacilli was poisonous to living bacilli, and concluded that by introducing it into the circulation he would destroy the bacilli that are said to exist with tuberculosis, and thereby cure the patient. It was ingenious enough upon the basis of his theory, but if this theory was not founded upon close-fitting facts, then his seeming ingenuity must lose its force and his theory must inherit the common legacy of all conjectures founded on false premises. Unfortunately for Dr. Koch he allowed the shout of triumph to be raised before he had gotten out of the woods. He has been, no doubt, an industrious investigator, and deserves credit for that much; and, morevoer, if he had discovered and brought to the knowledge of the medical profession a practicable cure for consumption, he would deserve all the badges of honor and all the titles of distinction that could be conferred upon him. But such bestowments as these should be made with discretion. They should not be showered upon heads and bosoms that have not deserved them. This would be to disparage their value and to discourage the meritorious. Many an humble country physician, whose fame has not extended more than fifty miles from his centre of work, has done more for the saving of human life and the mitigation of human suffering than some of the most renowned theorists of the profession. It might perhaps be germane here to inquire what practical benefit, what actual good, has resulted from the discovery of the comma bacillus and the cholera bacillus? Have there been more cases cured or have there been fewer victims to the encroachments of these diseases? If not, then *qui bono?* Of what good is it and where is the place for the throwing up of hats? Dr. Koch's researches may serve to forge a link in the golden chain, although I doubt it, but there will have to be more links added before the chain is long enough to reach the goal. Of course microphobists (for such have many become) will sneer at this, but let them laugh who win in the end. They (the microphobists) really seem to be laying the mines for their own destruction, and it even seems probable they will sooner or later spring them upon themselves. To all such I would commend the perusal of an article on "Professional Fanaticism" in the *North American Practitioner*, Vol. 2, No. 11. Among other things, he (Dr. Newomar) mentions a fact that I have been gathering from the reports of others, that pneumonia is more fatal to-day than it was thirty or forty years ago. I have been noticing this for years. Most of our prominent men die of pneumonia, and whenever it is announced that the distinguished Mr. so-and-so is sick of pneumonia, another announcement soon follows that he is dead. It has gotten to be so that I invariably expect this second announcement after the first. Indeed, in this day of "advanced thought" and "marvellous progress," in this last quarter of the nineteenth century the treatment of the acute phlegmasiæ has become sadly retrograde and fearfully unsuccessful. This ought not to be. It is a standing disgrace to the profession, and ought to be wiped out; but it will never be wiped out as long as the profession throw away common sense methods and rational treatment that long have stood the test of experience, and join in the chase, the wild hunt, after microbes, fine-spun theories and other such immaterial foolishness.

It is unfortunate for the sick that bacteriology was ever so enthroned in the mind of the profession. It afforded such a chance for hobby riding, and doctors do love to ride hobbies, oh, amazingly! Unfortunately, ever since its enthronement and ever since the assertion by Dr. Austin Flint that pneumonia was a limited disease, or had a certain course to run, and was an infectious disease, the poor victim of pneumonia has had to pay the penalty, even more than before, with his life. Ever since, even more than before, the treatment has gone crooked. I believe the theories of Dr. Koch and Dr. Flint have both been deleterious. I have been practicing medicine about forty-seven years, and I can remember many cases of pneumonia and acute bronchitis and other phlegmasiæ recovered then, and I can see from the reports how they die now—not from change of type, but from change of treatment. It distresses me.

There is no royal road to recovery from an acute phlegmasia, and I would advise all young practitioners, if they have Dr. Flint's practice, after noticing what he says on diagnosis and pathology, to close the book and lay it away out of sight, or else carefully cut out and burn what pertains to treatment, especially of pneumonia. So much absorbing interest devoted to microscopic studies and so much noise made about the infinitesimal serve to direct the minds of practitioners from rational and established methods, while the theories that result are on a par with the magnitude of the objects discovered. This last is said, not of Dr. Flint, but of the tendency of the times.

